# Hemichorea Associated With Nigrostriatal Dysfunction: Case Report of a Patient With an Ipsilateral Infarct in the Lenticular Nucleus and Internal Capsule

**DOI:** 10.1155/crnm/6054686

**Published:** 2025-06-17

**Authors:** Makoto Kobayashi

**Affiliations:** Department of Neurology, Asahi General Hospital, 1326 I, Asahi, Chiba 289-2511, Japan

## Abstract

Hemichorea is a rare manifestation of ischemic stroke whose lesion is typically located in the contralateral basal ganglia. Its pathomechanism has not been elucidated completely; however, it may be related to nigrostriatal dysfunction. In patients with hemichorea, dopamine transporter-single photon emission computed tomography (DAT-SPECT) reportedly displayed decreased tracer accumulation in the contralateral striatum. Moreover, in exceptional cases, responsible lesions were located in the ipsilateral cerebral hemisphere. This case report describes an 84-year-old man who presented with three weeks of intermittent, involuntary, and twisting movements in his right limbs. On physical examination, the patient had right-sided hemichorea without other neurological deficits. The choreic movements were more frequent in the lower limb than in the upper and provoked when he tried to take a certain posture or engaged in mental arithmetic. Magnetic resonance imaging performed on suspicion of stroke detected a right hemispheric subacute infarct in the posterior part of the lenticular nucleus and posterior limb of the internal capsule. Furthermore, DAT-SPECT revealed decreased tracer accumulation in the right striatum. He was administered oral antiplatelet medication after being diagnosed with lacunar infarction. The choreic movements gradually reduced over the next 8 months and eventually disappeared. The lesion in the lenticular nucleus and internal capsule was considered to have induced ipsilesional hemichorea, considering the temporal proximity between the hemichorea and ischemic stroke. Although DAT-SPECT findings in patients with ipsilesional hemichorea have not been reported, this case suggests that nigrostriatal dopamine dysfunction can contribute to the pathogenesis of ipsilesional hemichorea.

## 1. Introduction

Chorea is a hyperkinetic disorder that entails involuntary, nonrhythmic, and flowing movements in the face, neck, trunk, or limbs [1]. Hemichorea indicates a condition in which chorea is unilaterally observed and is often caused by ischemic or hemorrhagic stroke in the basal ganglia. Other relatively common causes include diabetic striatopathy due to hyperglycemia, Sydenham's disease, polycythemia vera, and chorea gravidarum [1, 2].

The lesion responsible for hemichorea is traditionally located in the contralateral subthalamic nucleus (STN). The presence of lesions in the cerebral cortex, caudate nucleus, putamen, globus pallidus, or thalamus can also evoke contralesional hemichorea [3–6]. In addition, in exceptional cases, patients demonstrate hemichorea with lesions on the ipsilateral side. The causes of ipsilesional hemichorea include tumors, hemorrhages, and infarcts in the unilateral cerebral hemisphere. In ischemic stroke, it was found with lesions of the frontal cortex [7], STN [8], or thalamus [9].

Hemichorea is currently hypothesized to result from dysfunction of the hyperdirect and/or indirect pathways in the contralateral basal ganglia [10]. Although its pathogenesis has not been completely elucidated, recent advances in functional imaging provide useful findings that help to understand the mechanism [11]. Especially, on dopamine transporter-single photon emission computed tomography (DAT-SPECT), the severity of chorea was reported to be associated with the decrease in putaminal tracer uptake in patients with Huntington's disease [12]. Moreover, a unilateral decrease in striatal tracer accumulation was associated with contralateral hemichorea in patients with diabetic striatopathy [13]. In patients with choreoacanthocytosis or polycythemia vera who had generalized chorea with a right–left difference, striatal accumulation on the less symptomatic side was lower than that on the more symptomatic side [14, 15].

Herein, we report the rare case of a patient with ipsilesional hemichorea in whom magnetic resonance imaging (MRI) detected a small ischemic lesion in the lenticular nucleus and internal capsule. Moreover, DAT-SPECT revealed decreased tracer accumulation in the striatum of the symptomatic side.

## 2. Case Presentation

An 84-year-old right-handed man visited our hospital because he experienced intermittent, involuntary, and twisting movements in his right limbs for three weeks. Although the frequency of the spontaneous movements was decreasing, these movements sometimes occurred when he used chopsticks at meals or when he tried to stand up. It was not apparent whether the onset of the movements was sudden or insidious. He had a medical history of diabetes mellitus, gastric cancer that was successfully resected 5 years earlier without recurrence, and reflux esophagitis. Head trauma or neurosurgical procedures were not mentioned in the anamnesis. His familial history was unremarkable and did not include neurodegenerative diseases. The patient's oral medications were saxagliptin 5 mg/day, mitiglinide calcium 30 mg/day, and mosapride citrate 15 mg/day. On physical examination, intermittent choreic movements were observed in his right upper and lower extremities. The movements predominantly appeared in the lower extremity of the affected side and became more apparent when he tried to adopt a certain posture or performed mental arithmetic (Supporting video (available here)). He did not have motor paresis or sensory disturbance in the face and limbs. The finger-tapping test revealed no abnormality, and his gait was normal in the absence of choreic movements.

**Figure 1:**
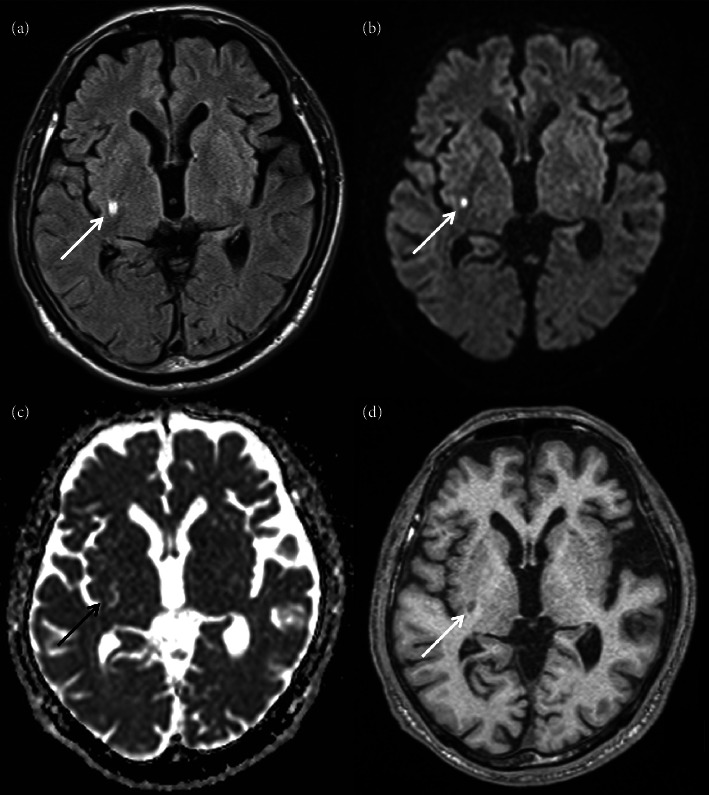
Head magnetic resonance imaging findings. Fluid-attenuated inversion recovery imaging revealed a hyperintensity lesion in the right lenticular nucleus and internal capsule (arrow, (a)). The lesion was partly hyperintense on diffusion-weighted imaging (arrow, (b)) and corresponded to an isointense area accompanied by an adjacent irregular hyperintensity on apparent diffusion coefficient map (arrow, (c)). Moreover, on T1 images, it was largely hypointense (arrow, (d)) in the absence of striatal hyperintensities.

MRI was performed on suspicion of stroke. Fluid-attenuated inversion recovery imaging revealed a high-intensity lesion in the posterior part of the right lenticular nucleus and posterior limb of the right internal capsule (arrow, Figure 1(a); greatest diameter on the axial plane, 6.6 mm). The lesion was partly hyperintense on diffusion-weighted imaging (arrow, Figure 1(b); 4.5 mm) and corresponded to an isointense area accompanied by an irregular hyperintensity on apparent diffusion coefficient map (arrow, Figure 1(c); unmeasurable due to the isointense lesion). On T1 images, it was largely hypointense (arrow, Figure 1(d); 7.1 mm) with no striatal hyperintensities. Magnetic resonance angiography detected no apparent stenosis in the major cerebral arteries. His routine blood test results were unremarkable except for the triglyceride (158 mg/dL; normal range: 30–149 mg/dL), hemoglobin A1c (6.3%; 4.6%–6.2%), and random blood glucose (179 mg/dL; 70–140 mg/dL) levels. Chest x-ray and electrocardiography findings were unremarkable. He was diagnosed with lacunar cerebral infarction in the subacute phase and treated with oral clopidogrel 75 mg/day and lansoprazole 15 mg/day. Symptomatic treatment for choreic movements was not started since the involuntary movements did not substantially affect the patient's daily life and were regressing.

**Figure 2:**
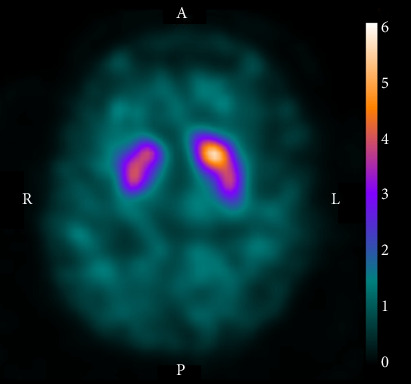
Dopamine transporter-single photon emission computed tomography findings. The tracer accumulation was decreased in the right striatum. The scale bar indicates the extent of tracer accumulation when the mean background concentration is postulated as 1.

DAT-SPECT with ^123^I-N-ωfluoropropyl-2β-carbomethoxy-3β-(4-iodophenyl)nortropane (^123^I-FP-CIT) was subsequently performed for hemichorea scrutiny. It revealed decreased tracer accumulation in the right striatum (Figure 2). The specific binding ratio was 4.19 for the right striatum and 5.14 for the left, with an asymmetry index of 20.3% (normal range: < 10%). Additional blood test results, including ammonia, lupus anticoagulant, antinuclear antibody, thyroid-stimulating hormone, free triiodothyronine, free thyroxine, anticardiolipin antibody (immunoglobulin G and M subclasses), and antiSmith antibody levels were normal. Electroencephalography revealed no abnormal findings. On follow-up visits to our clinic, the choreic movements waned over the next 8 months and eventually disappeared. Moreover, the patient did not display Parkinsonism for another 2 years.

## 3. Discussion

In the presented case, a small lesion in the lenticular nucleus and internal capsule was considered to have caused ipsilesional hemichorea because of the temporal proximity between the hemichorea and ischemic stroke. Moreover, DAT-SPECT revealed decreased tracer accumulation in the striatum of the symptomatic side.

Hemichorea, a rare manifestation of ischemic stroke, is usually caused by lesions of the lenticular nucleus, STN, or cerebral cortex on the contralateral side [3]. The involuntary movements are often provoked by volitional movements or mental stress as observed in the current patient. However, in exceptional cases, responsible lesions were reportedly located on the ipsilateral side of hemichorea. For example, a 23-year-old woman underwent a right pterional craniotomy for pituitary adenoma and demonstrated right-sided hemichorea and hemiballism on the second postoperative day [16]. Head MRI indicated a large acute infarct in the territory of the right internal carotid artery, involving a large part of the right frontal, temporal, and parietal cortex and a portion of the right basal ganglia. In another patient, a 72-year-old woman who presented with left-sided hemichorea, an acute ischemic lesion in the left hemisphere was found to extend from the thalamus to the cerebral peduncle [9]. In addition, a 63-year-old man was also reported to present with hemichorea and hemiballism in whom MRI detected a small infarct in the ipsilateral STN [8]. Left-sided hemichorea was observed in a 64-year-old woman with an old lesion in the right frontal lobe and acute infarcts in the territory of the left anterior cerebral artery [7]. These reports indicate cerebral lesions that are at least seemingly not restricted to a specific location can provoke ipsilesional hemichorea.

**Figure 3:**
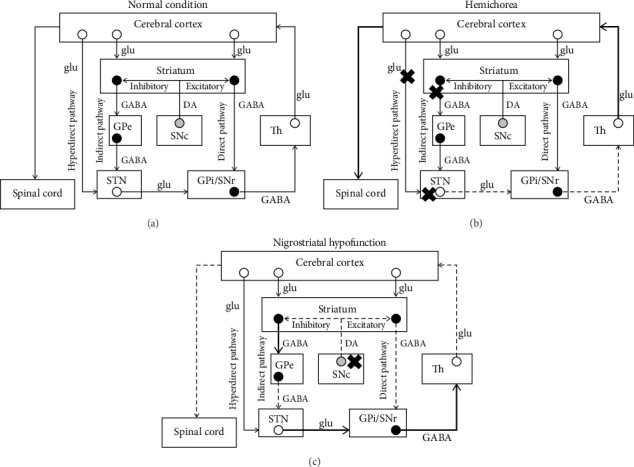
Illustrations of basic circuits for the cerebral cortex, basal ganglia, and thalamus ((a) normal condition; (b) pathological condition in patients with hemichorea; and (c) pathological condition in patients with nigrostriatal hypofunction). There are three important pathways from the cerebral cortex to the GPi/SNr (a), which are the hyperdirect pathway (cortex–STN–GPi/SNr), direct pathway (cortex–striatum–GPi/SNr), and indirect pathway (cortex–striatum–GPe–STN–GPi/SNr). When a voluntary movement is performed, inputs from the cerebral cortex initially suppress the thalamus extensively via the hyperdirect pathway. Subsequently, through the direct pathway, the inputs focally disinhibit the thalamus, which is otherwise under strong suppression by GABAergic projections from the GPi/SNr. Finally, the inputs from the cortex suppress the thalamus extensively again via the indirect pathway. Considering the glutamatergic neurons in the thalamus project excitatory fibers to the cortex, the focal disinhibition of the thalamus induces the excitatory effects on the cortex and is associated with the voluntary movement; meanwhile, the extensive suppression of the thalamus helps to eliminate unnecessary movements. Therefore, the disruption of the hyperdirect or indirect pathway may lead to unnecessary movements (i.e., hemichorea or hemiballism (b)). Furthermore, nigrostriatal hypofunction can result in insufficient disinhibition of the thalamus and hypokinesia because dopaminergic projections from the SNc to the striatum promote the direct pathway and suppress the indirect pathway (c). Lesions are indicated using X marks. The bold and broken arrows indicate increased and decreased neural activity, respectively. Open circles: glutamatergic excitatory neurons; closed circles (black): gamma-aminobutyric acidergic inhibitory neurons; closed circles (gray): dopaminergic neurons; DA: dopamine; GABA: gamma-aminobutyric acid; glu: glutamic acid; GPe: globus pallidus externa; GPi: globus pallidus interna; SNc: substantia nigra pars compacta; SNr: substantia nigra pars reticulata; STN: subthalamic nucleus; Th: thalamus.

The mechanism of chorea has not been completely elucidated; however, some major hypotheses can be found in the literature. The basal ganglia consist of the striatum, globus pallidus interna and externa (GPi and GPe), STN, and substantia nigra pars compacta and reticulata (SNc and SNr). According to a major hypothesis, there are three important pathways when processing information from the cerebral cortex [10] (Figure 3(a)). They are the hyperdirect pathway (constituents: cortex–STN–GPi/SNr), direct pathway (cortex–striatum–GPi/SNr), and indirect pathway (cortex–striatum–GPe–STN–GPi/SNr). During a voluntary movement, inputs from the cortex initially suppress the thalamus extensively through the hyperdirect pathway, subsequently causing focal excitation of the thalamus via the direct pathway, and finally suppressing the thalamus extensively again via the indirect pathway. The focal excitation of the thalamus excites the cortex and is associated with the voluntary movement, while the extensive suppression of the thalamus is considered a requirement to suppress unnecessary movements. Therefore, STN hypofunction can induce dysfunction in the hyperdirect and indirect pathways, leading to insufficient suppression of unnecessary movements and contralateral hemichorea/hemiballism [10] (Figure 3(b)). Moreover, given that dopaminergic projections from the SNc to the striatum promote the direct pathway and suppress the indirect pathway, nigrostriatal hypofunction may result in insufficient excitation of the thalamus and hypokinesia of the contralateral side [10] (Figure 3(c)).

Despite the unapparent causal association between dopaminergic dysfunction and hemichorea, DAT-SPECT was reported to detect decreased tracer accumulation in the contralateral striatum. An 82-year-old man with hyperglycemia (blood glucose level: 590 mg/dL) presented with left-sided hemichorea and hemiballism. MRI displayed a T1-weighted hyperintense lesion in the right putamen. Moreover, on DAT-SPECT, decreased tracer accumulation was found in the right striatum [13]. In a 33-year-old man with chorea-acanthocytosis who demonstrated asymmetrical choreic movements with left-sided predominance, DAT-SPECT revealed less tracer accumulation in the right striatum than in the left [14]. A 70-year-old woman with polycythemia vera demonstrated chorea of the four limbs with right-sided predominance. DAT-SPECT revealed bilateral decreased accumulation that was more severe in the left striatum [15]. Although decreased striatal accumulation has been reported in the cases of contralesional hemichorea, DAT-SPECT findings in ipsilesional hemichorea have not been reported.

The mechanism of ipsilesional hemichorea remains elusive; however, some speculations have been introduced in previous articles. In an article, patients with ipsilesional hemichorea were speculated to have the congenital uncrossed pyramidal tract or active uncrossed pyramidal tract [7]. Another speculation is that abnormal activity in the damaged cerebral hemisphere might have been channeled to the contralateral hemisphere via the corpus callosum, resulting in ipsilesional hemichorea [16]. Alternatively, lesions on the ipsilateral side of hemichorea could have influenced brain organs that were able to affect bilateral limbs or were connected to bilateral motor-related neural structures. The possible brain organs previously described were the pedunculopontine nucleus [8], secondary motor area [7], or subthalmic nucleus [8, 16]. Other speculations include unilateral lesions that cause bilateral chorea and contralateral hemiplegia with resultant ipsilateral hemichorea, those that have mass effect and compress the hyperdirect and/or indirect pathways in the contralateral hemisphere, and those that decrease functional activity of the contralateral hyperdirect and/or indirect pathways as a result of diaschisis. In the current patient, DAT-SPECT findings suggested right nigrostriatal hypofunction probably due to ischemia of the nigrostriatal tract and/or striatum. Considering the abovementioned hypothesis, the patient would have demonstrated left-sided hypokinesia; however, he had hyperkinesia (i.e., hemichorea) in the right limbs and did not have hypokinesia. Although the reason for these neurological findings is unclear, it is possible that some adaptive changes in the brain structures that were able to affect bilateral limbs counteracted the hypokinesia and simultaneously gave rise to hemichorea.

The current patient was a diabetic and had elevated levels of hemoglobin A1c and random blood glucose; therefore, diabetic chorea was included in the differential diagnosis. In addition, as described above, patients with diabetic chorea reportedly had decreased tracer uptake in the striatum on DAT-SPECT. However, hemoglobin A1c and random blood glucose levels of the presented case were low as compared with those reported in patients with diabetic chorea [2, 13]. The mildly elevated hemoglobin A1c and random blood glucose levels and absence of T1 striatal hyperintensities on MRI suggested that the current patient's hemichorea was not caused by diabetic striatopathy.

In this case presentation, the patient was diagnosed as having lacunar infarction and treated with oral clopidogrel. The ischemic MRI lesion was less than 20 mm in greatest diameter and located in the territory of the right basal penetrating arteries, accompanied by no apparent stenosis in the trunk and major branches of the right middle cerebral artery. These findings indicated the ischemic lesion was classified as lacunar infarction [17]; however, echocardiogram should have been performed to definitely exclude cardioembolic sources. Since the patient's symptoms gradually improved over 3 weeks on the first visit to our hospital, the antiplatelet medication was initiated for the secondary prevention of ischemic stroke.

The mechanism of ipsilesional hemichorea has not been elucidated possibly because of its rarity. Therefore, accumulation of case reports may be important and lead to the better understanding of this infrequent chorea. A major limitation of this report is that more cases with ipsilesional chorea are necessary to obtain robust conclusions regarding the DAT-SPECT findings. In summary, this case report has demonstrated that hemichorea can be observed with an ipsilateral cerebral lesion. Moreover, the observed involuntary movements may be associated with ipsilateral nigrostriatal dopamine dysfunction.

## Data Availability

All data generated or analyzed during this study are included within the article and are available from the corresponding author upon reasonable request.

## Supporting Information

Supporting video legend: The patient was initially sitting on a chair with his hands on the thighs, and then he raised his upper limbs. When he tried to keep his upper limbs parallel to the floor, distal-dominant and involuntary movements appeared in the right upper limb. In the next scene, involuntary movements were observed in his right lower limb. The patient intermittently tapped his right foot against the floor or swayed his right leg while he engaged in mental arithmetic.
